# Assessing the predictive value of smoking history for immunotherapy outcomes in bladder cancer patients

**DOI:** 10.3389/fimmu.2024.1404812

**Published:** 2024-06-13

**Authors:** Jianqiu Kong, Yitong Zou, Hua Zhou, Yi Huang, Ying Lin, Shuogui Fang, Zhijian Chen, Junjiong Zheng, Yaqiang Huang, Zefeng Shen, Weibin Xie, Xinxiang Fan

**Affiliations:** ^1^ Department of Urology, Sun Yat-Sen Memorial Hospital, Sun Yat-Sen University, Guangzhou, Guangdong, China Guangdong Provincial Key Laboratory of Malignant Tumor Epigenetics and Gene Regulation, Sun Yat-Sen Memorial Hospital, Sun Yat-Sen University, Guangzhou, Guangdong, China; ^2^ Department of Urology, Pu ‘er People’s Hospital of Yunnan Province, Pu’er, Yunnan, China; ^3^ Department of Endocrinology, Sun Yat-Sen Memorial Hospital, Sun Yat-Sen University, Guangzhou, Guangdong, China; ^4^ Department of Radiotherapy, Sun Yat-Sen Memorial Hospital, Sun Yat-Sen University, Guangzhou, Guangdong, China; ^5^ Department of Urology, Zhongshan City People’s Hospital, Sunwen East Road, Zhongshan, Guangdong, China; ^6^ Department of Urology, Peking University Shenzhen Hospital, Shenzhen, Guangdong, China

**Keywords:** bladder cancer, immunotherapy, smoking history, PD-L1 expression, clinical outcomes

## Abstract

**Background:**

The therapeutic effectiveness of immune checkpoint inhibitors (ICIs) in bladder cancer varies among individuals. Identifying reliable predictors of response to these therapies is crucial for optimizing patient outcomes.

**Methods:**

This retrospective study analyzed 348 bladder cancer patients treated with ICIs, with additional validation using data from 248 patients at our institution who underwent PD-L1 immunohistochemical staining. We examined patient smoking history, clinicopathological characteristics, and immune phenotypes. The main focus was the correlation between smoking history and immunotherapy outcomes. Multivariate logistic and Cox proportional hazard regressions were used to adjust for confounders.

**Results:**

The study cohort comprised 348 bladder cancer patients receiving ICIs. Among them, 116 (33.3%) were never smokers, 197 (56.6%) were former smokers (median pack-years = 28), and 35 (10.1%) were current smokers (median pack-years = 40). Analysis revealed no statistically significant difference in overall survival across different smoking statuses (objective response rates were 11.4% for current smokers, 17.2% for never smokers, and 22.3% for former smokers; *P* = 0.142, 0.410, and 0.281, respectively). However, a notable trend indicated a potentially better response to immunotherapy in former smokers compared to current and never smokers. In the validation cohort of 248 patients from our institution, immunohistochemical analysis showed that PD-L1 expression was significantly higher in former smokers (55%) compared to current smokers (37%) and never smokers (47%). This observation underscores the potential influence of smoking history on the tumor microenvironment and its responsiveness to ICIs.

**Conclusion:**

In conclusion, our study demonstrates the importance of incorporating smoking history in predicting the response to immunotherapy in bladder cancer patients, highlighting its role in personalized cancer treatment approaches. Further research is suggested to explore the comprehensive impact of lifestyle factors on treatment outcomes.

## Introduction

Bladder cancer, particularly in its advanced stages, represents a significant clinical challenge and is a major public health concern (1). The introduction of immune checkpoint inhibitors (ICIs) has revolutionized the therapeutic landscape for this malignancy, signifying a major advancement in oncology (2). ICIs, by harnessing the body**’**s immune system to target and destroy cancer cells, have offered new hope for patients with advanced bladder cancer. Despite their transformative potential, ICIs have shown varied effectiveness across the patient population, revealing an essential gap in our understanding of how best to predict and optimize treatment responses (3). Recent studies have demonstrated that combinations of ICIs with platinum-based chemotherapy or with another ICI in the first-line setting for patients with metastatic urothelial carcinoma have mixed results, but are associated with a survival benefit, highlighting the nuanced impact of treatment combinations (4). Moreover, our previous research has extensively analyzed the complex interactions within the tumor microenvironment and mechanisms of immune evasion, significantly influencing the efficacy of anti-PD-1/PD-L1 and anti-CTLA-4 therapies. This bibliometric study, leveraging a comprehensive analysis of literature spanning over two decades, highlights the importance of understanding these dynamics in the context of tumor immunotherapy (5). Furthermore, the impact of patients**’** performance status on oncologic outcomes when treated with ICIs has been evaluated, showing that even patients with poorer performance status may benefit from ICIs compared to chemotherapy, though outcomes vary significantly with the degree of performance status (6). This variability highlights the critical need for reliable biomarkers that can guide therapeutic decisions and improve patient outcomes.

The utility of programmed death ligand-1 (PD-L1) expression as a biomarker for ICIs in bladder cancer has been a focal point of research. Despite its potential, the clinical application of PD-L1 expression in bladder cancer remains nuanced and complex (7). Furthermore, the tumor mutation burden (TMB), traditionally used as a predictor of ICI responsiveness, reflects the total number of mutations within a tumor genome (8). This metric has been linked to immune recognition through the generation of neoantigens, which are novel peptides that the immune system can target (9).

However, our study introduces the concept of Neoantigen Mutation Burden (NMB) as a potentially more direct measure of neoantigen production than TMB. Unlike TMB, which counts all mutations, NMB specifically quantifies those mutations likely to produce neoantigens that can stimulate an immune response. This distinction is crucial because not all mutations contribute to neoantigen production, and hence, NMB might provide a more precise prediction of the immunotherapy response in bladder cancer.

In this intricate context, our study turns its attention to smoking history, a well-documented risk factor for bladder cancer. Previous research in other cancers suggests a correlation between smoking and higher NMB, raising the possibility of its influence on ICI efficacy (10–12). Notably, an analysis of the IMvigor 210 cohort indicated a lack of correlation between smoking status and mutational burden in bladder cancer, challenging the straightforward application of NMB as a predictor (13). Furthermore, emerging research in other malignancies, such as renal cell carcinoma, has identified novel mechanisms and biomarkers, such as circPPAP2B, which controls metastasis through complex molecular interactions, highlighting the potential for cross-cancer insights that might also benefit bladder cancer research (14). This insight drives our investigation into the potential of detailed smoking history as a more accessible and possibly consistent predictor for ICIs in bladder cancer.

Our study aims to conduct an in-depth analysis of smoking history, quantified in terms of pack-years, alongside the exploration of PD-L1 expression levels in our own patient cohort. We seek to assess the predictive impact of smoking history on objective response rate (ORR), progression-free survival (PFS), and overall survival (OS) in bladder cancer patients treated with ICIs. This approach intends to complement the existing biomarkers, offering a broader, more accessible means of predicting treatment outcomes in a field where standard biomarkers like NMB present significant challenges.

## Methods

### Clinical samples and study population

This study included bladder cancer patients from two sources: the IMvigor 210 correlative research study cohort, who received ICI monotherapy, and a separate cohort of 248 patients from Sun Yat-sen Memorial Hospital of Sun Yat-sen University. For the IMvigor 210 cohort, clinicopathological data, including age, gender, detailed smoking history, intravesical BCG administration, baseline Eastern Cooperative Oncology Group Performance Status (ECOG Score), NMB and metastatic disease status, were collected. Smoking status was categorized into never smokers, former smokers, and current smokers, with pack-years quantified. We excluded patients with prior malignancies, autoimmune diseases, or incomplete medical records to ensure a homogenous study population focused solely on bladder cancer immunotherapy outcomes. For the Sun Yat-sen Memorial Hospital cohort, we specifically focused on PD-L1 immunohistochemical staining in 248 bladder cancer patients.

### NMB assessment and PD-L1 testing

Sample collection and DNA extraction protocols, as previously described, were applied. NMB was calculated from the Dana-Farber Cancer Institute OncoPanel next-generation sequencing platform. PD-L1 tumor proportion score (TPS) in the IMvigor 210 cohort was measured using antibodies like 22C3, SP263, and E1L3N, represented as the percentage of tumor cells showing positive staining (15). In the Sun Yat-sen Memorial Hospital cohort, PD-L1 expression was evaluated immunohistochemically and graded on a scale from 0 to 3, reflecting the intensity and proportion of positive tumor cell staining.

### Clinical outcomes

The objective response rate (ORR) and overall survival (OS) were evaluated using the Response Evaluation Criteria in Solid Tumors version 1.1. ORR encompassed complete or partial response rates, while OS was measured from the start of treatment to disease death.

### Statistical analysis

Continuous and categorical variables were compared using the Wilcoxon rank sum test and Fisher exact test. Kaplan-Meier methods estimated time-to-event distributions, with log-rank tests for differences between groups. Cox proportional hazards regression models assessed the relationship between clinical outcomes (OS) and variables such as smoking history, NMB, and PD-L1 TPS, following the proportional hazards assumption confirmed through Schoenfeld residuals. Multivariable analyses adjusted for factors like gender, immune phenotype, and baseline ECOG Score. Statistical tests were two-sided, with a 95% confidence interval and a significance level set at 0.05.

## Results

### Study population from IMvigor 210 cohort

**Figure 1:**
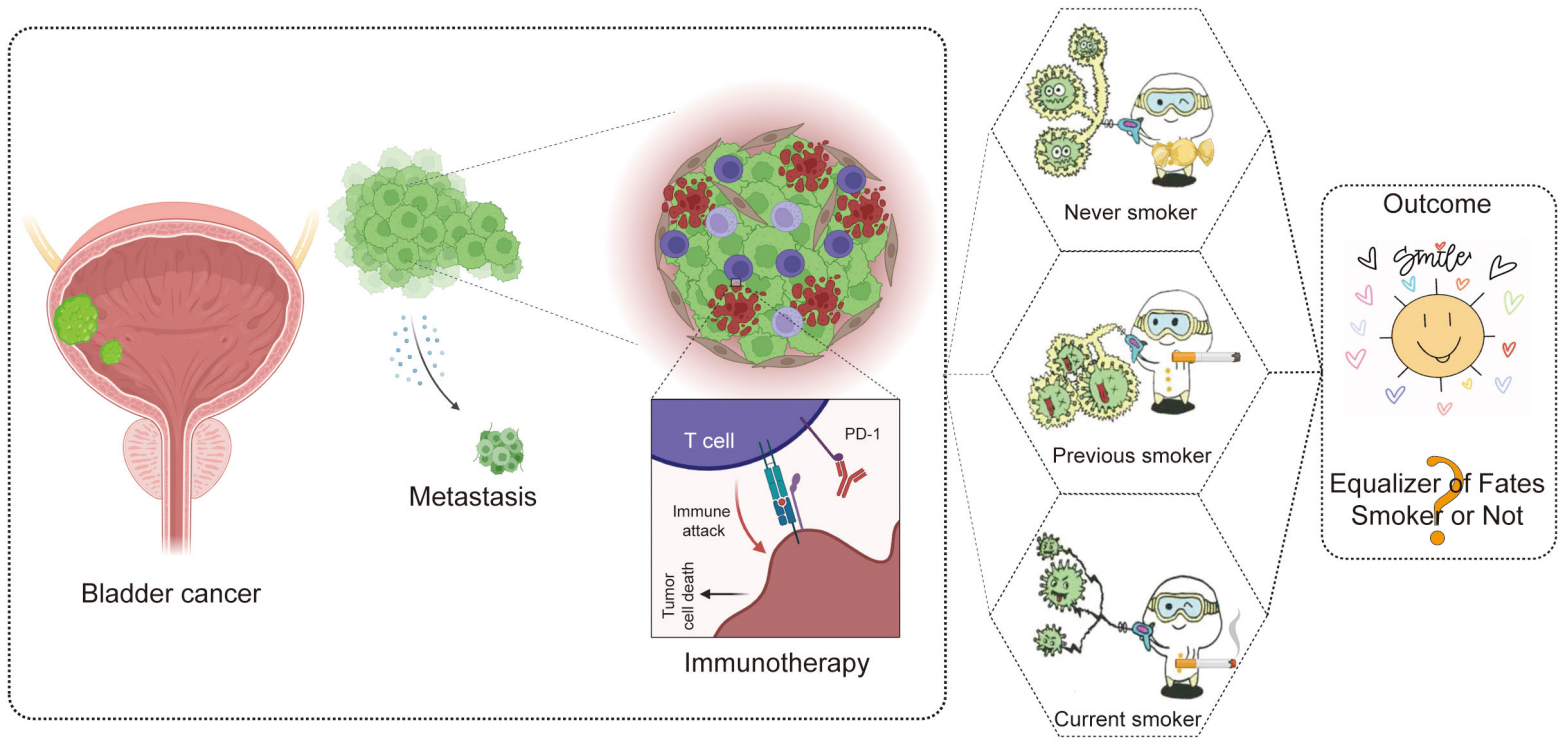
Flow chart of the analyses performed in this study.

Our study identified a total of 348 bladder cancer patients treated with immunotherapy monotherapy, categorized into never smokers (116, 33.3%), former smokers (197, 56.6%; median pack-years = 28), and current smokers (35, 10.1%; median pack-years = 40). Across these groups, the distribution of immune phenotype, intravesical BCG administration, metastatic disease status, and baseline ECOG Score was similar (**Table 1**). In **Figure 1**, our analysis of PD-L1 therapy in metastatic bladder cancer patients is presented. We observed no significant correlation between smoking status and overall survival in those undergoing immunotherapy. Intriguingly, former smokers showed better response to treatment compared to current or non-smokers. This highlights the potential impact of smoking history on immunotherapy efficacy and warrants further investigation. Best objective responses were observed in 17.2%, 22.3%, and 11.4% of never, former, and current smokers, respectively (**Supplementary Table 1**).

**Table 1:** Baseline clinicopathological characteristics (N = 348).

Variable	Overall, N = 348^1^	CURRENT, N = 35^1^	NEVER, N = 116^1^	PREVIOUS, N = 197^1^	*P*-value^2^
Sex					0.025
Female	76 (22%)	5 (14%)	35 (30%)	36 (18%)	
Male	272 (78%)	30 (86%)	81 (70%)	161 (82%)	
Immune phenotype					0.200
Desert	76 (27%)	9 (27%)	31 (34%)	36 (22%)	
Excluded	134 (47%)	16 (48%)	41 (46%)	77 (48%)	
Inflamed	74 (26%)	8 (24%)	18 (20%)	48 (30%)	
NA	64	2	26	36	
Intravesical BCG administered					0.700
No	265 (76%)	27 (77%)	91 (78%)	147 (75%)	
Yes	83 (24%)	8 (23%)	25 (22%)	50 (25%)	
Baseline ECOG Score					0.500
0	134 (39%)	12 (34%)	44 (38%)	78 (40%)	
1	196 (56%)	19 (54%)	68 (59%)	109 (55%)	
2	18 (5.2%)	4 (11%)	4 (3.4%)	10 (5.1%)	
Met Disease Status					0.700
Liver	98 (31%)	8 (23%)	34 (32%)	56 (32%)	
LN Only	60 (19%)	7 (20%)	23 (22%)	30 (17%)	
Visceral	158 (50%)	20 (57%)	49 (46%)	89 (51%)	
NA	32	0	10	22	
Received platinum					0.014
No	76 (22%)	4 (11%)	18 (16%)	54 (27%)	
Yes	272 (78%)	31 (89%)	98 (84%)	143 (73%)	
**Neoantigen burden per MB**	0.92 (0.47, 1.73)	0.88 (0.51, 1.92)	0.79 (0.35, 1.32)	1.03 (0.57, 1.80)	0.068
NA	103	10	42	51	
Lund2					0.991
Basal/SCC-like	66 (19%)	6 (17%)	20 (17%)	40 (20%)	
Genomically unstable	70 (20%)	6 (17%)	20 (17%)	44 (22%)	
Infiltrated	92 (26%)	9 (26%)	34 (29%)	49 (25%)	
UroA	102 (29%)	12 (34%)	38 (33%)	52 (26%)	
UroB	18 (5.2%)	2 (5.7%)	4 (3.4%)	12 (6.1%)	

^1^n (%); Median (IQR).

^2^Pearson’s Chi-squared test; Fisher’s exact test; Kruskal-Wallis rank sum test.

### Association between smoking history and clinical outcomes in IMvigor 210 cohort

**Figure 2:**
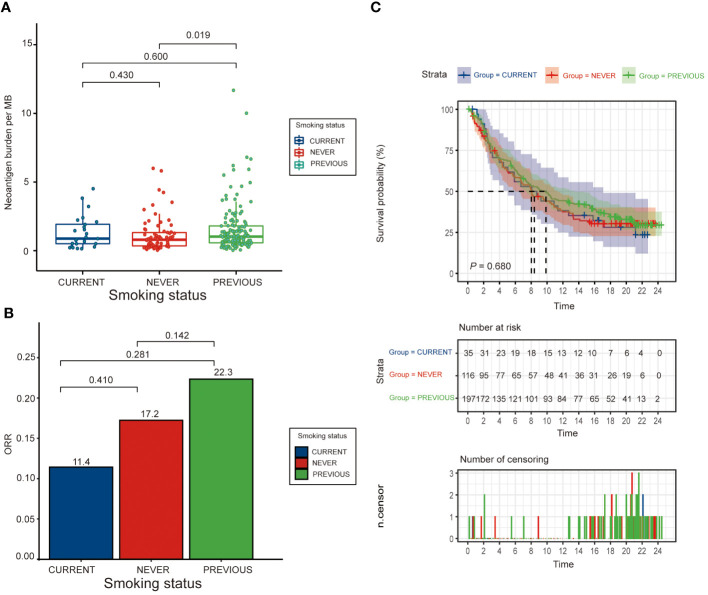
Clinical outcomes by smoking status. **(A)** Bar graph showing the distribution of neoantigen burden (measured in mutations per megabase, MB) across never, former, and current smokers, illustrating how smoking history correlates with neoantigen production in bladder cancer. **(B)** Bar graph presenting the objective response rate (ORR) to immunotherapy across different smoking statuses, and **(C)** Kaplan-Meier survival curves comparing overall survival (OS) among never, former, and current smokers, highlighting potential differences in treatment efficacy based on smoking history.

The analysis revealed no significant correlation between smoking status and overall survival (**Figure 2A**, **C**). However, former smokers seemed to have a trend towards better responses compared to current and never smokers. The neoantigen burden differed across smoking subgroups, with the highest in former smokers (median = 1.03; range [0.57, 1.80]), but the differences were not statistically significant (P > 0.05). The ORR also did not show significant differences among the groups, with the highest observed in previous smokers (22.3%) (**Figure 2B** and **Supplementary Table 2**).

In **Supplementary Figure 1**, we present the relationship between neoantigen burden per megabase (NMB) in the tumor genome and the risk of response to immunotherapy. This graph delineates a complex, non-linear correlation. It illustrates that variations in the quantity of neoantigens per MB significantly impact the likelihood of a positive therapeutic response. Key inflection points and thresholds within this relationship are highlighted, underscoring the intricate nature of neoantigen burden as a predictive biomarker for immunotherapy efficacy.

Multivariate analysis showed that while smoking status was not a significant predictor of survival (P = 0.680), there were significant associations with ECOG Score, NMB, and metastatic disease type, indicating the complexity of factors affecting survival outcomes (**Supplementary Table 3**).

### Validation of clinical utility in IMvigor 210 cohort

**Figure 3:**
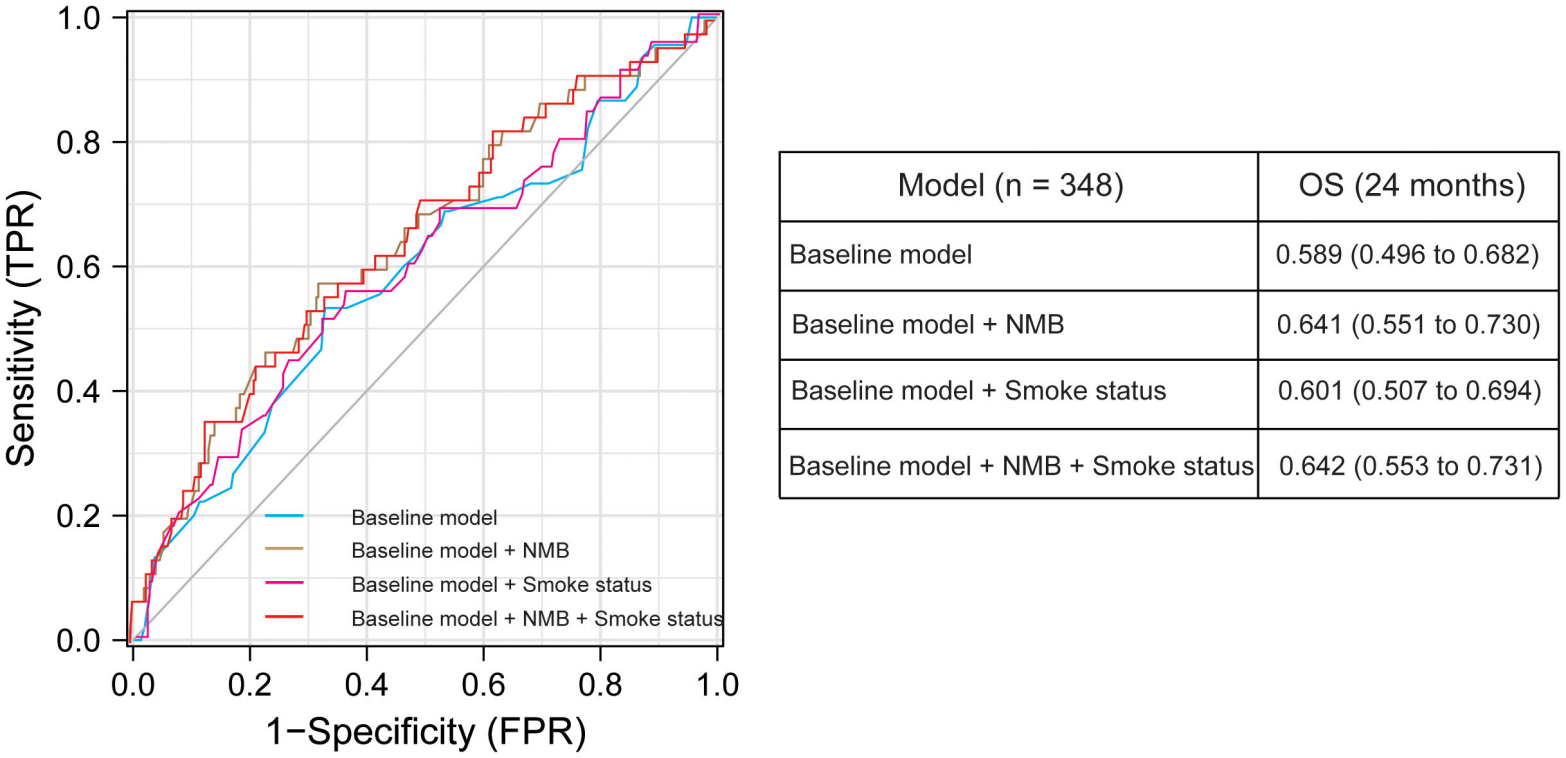
Graph showing the area under the curve (AUC) of the Receiver Operating Characteristic (ROC) for prediction models of 24-month overall survival (OS) using various clinical and molecular predictors. This figure includes data on sex, prior intravesical BCG therapy, ECOG performance status, Met expression, and TCGA molecular subtype. Performance status and lines of therapy were consistently included as covariates in all models. The table below the graph presents AUC values with 95% confidence intervals, derived from 500 bootstrap samples, providing insight into the robustness and reliability of our predictive models.

Incorporating smoking status into the predictive model enhanced its performance for overall survival (OS AUC = 0.601), demonstrating an additional value over the baseline model (OS AUC = 0.589). The model combining smoking status with NMB had a performance comparable to using NMB alone, suggesting the potential of smoking history as a surrogate for NMB (**Figure 3**). **Supplementary Figure 3** examines survival outcomes across various cancers based on smoking status, using data from the TCGA (The Cancer Genome Atlas) database. This figure features survival curves that contrast the prognoses of patients with different types of cancers, segmented by their smoking histories. A notable observation is the pronounced impact of smoking on survival outcomes in breast cancer patients, indicating a significant correlation between smoking and reduced survival in this subgroup. Conversely, for other cancers included in this analysis, the influence of smoking status on patient survival is comparatively less pronounced, suggesting variable impacts of smoking across different cancer types.

### External validation using Sun Yat-sen Memorial Hospital cohort

**Figure 4:**
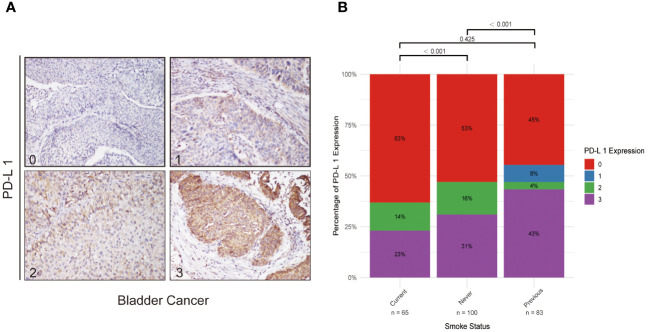
PD-L1 Expression in Bladder Cancer and Its Correlation with Smoking Status. **(A)** Series of immunohistochemical staining images of PD-L1 in bladder cancer tissues, with quantification of expression levels categorized on a scale from 0 (low) to 3 (high). These images exemplify the variability in PD-L1 expression potentially influenced by smoking status. **(B)** Bar graph demonstrating the variations in PD-L1 expression across never smokers, former smokers, and current smokers, offering insights into how smoking history might affect immune modulation in bladder cancer.

An external validation using data from 248 bladder cancer patients from Sun Yat-sen Memorial Hospital was conducted (**Figure 4**). This included 65 current smokers, 100 never smokers, and 83 former smokers. PD-L1 expression varied significantly across smoking statuses, with a higher proportion of high PD-L1 expression in former smokers. Significant differences in PD-L1 expression levels were observed between current and never smokers (P < 0.001), and between never and former smokers (P < 0.001), but not between current and former smokers (P = 0.425). This external cohort supports the initial findings from the IMvigor 210 cohort, underscoring the relevance of smoking history in the context of immunotherapy response.

## Discussion

In the context of bladder cancer, where the therapeutic efficacy of immune checkpoint inhibitors (ICIs) can vary significantly among individuals (16), our study provides a critical assessment of how smoking history might serve as an informative predictor of immunotherapy outcomes. By introducing the concept of Neoantigen Mutation Burden (NMB) as a measure distinct from the traditional Tumor Mutation Burden (TMB), our study addresses a notable gap in current oncological practices, particularly in the context of limited availability of established biomarkers such as TMB. By systematically evaluating the relationship between smoking history and the response to ICIs, our study not only underscores the importance of considering patient lifestyle factors in treatment planning but also contributes to the growing body of evidence supporting the need for personalized treatment strategies in oncology. Our findings suggest that smoking history could be a readily available, yet underutilized, indicator in predicting the efficacy of immunotherapy, offering valuable insights for optimizing patient outcomes in the treatment of bladder cancer.

### Smoking history: Beyond a risk factor

Traditionally, smoking history has been considered predominantly as a risk factor for cancer development (17). Our study, however, sheds light on its potential impact on the response to immunotherapy in bladder cancer, particularly in relation to NMB. The observation that former smokers might derive more benefit from ICIs than current or never smokers invites a reevaluation of smoking history in oncologic treatment planning. This could be attributed to the complex interaction between the carcinogenic effects of smoking and the heightened production of neoantigens (NMB) by the resultant tumors (18). Specifically, the mutagenic influence of smoking may induce a broader array of neoantigens, thereby potentially augmenting the efficacy of ICIs (19). Research by Alexandrov et al. supports this, showing that smoking increases the total number of somatic mutations in cancers, which likely leads to higher neoantigen production (20). Moreover, McGranahan et al. found that a high mutational burden, potentially induced by smoking, correlates with improved survival in lung cancer patients treated with immunotherapies, due to an increased neoantigen load (21). These findings imply a paradigm shift: smoking history might serve not just as an indicator of cancer risk, but also as a nuanced predictor of response to immunotherapies. Nevertheless, the broader implications of this relationship warrant further investigation, particularly in the context of long-term treatment outcomes and survival benefits. This highlights the need for a more nuanced consideration of smoking history in both risk assessment and therapeutic strategy development for bladder cancer.

### PD-L1 expression and smoking: A subtle association

Our investigation into the relationship between PD-L1 expression and smoking status uncovered a complex interplay, rather than a straightforward correlation. This complexity arises from potential confounding factors such as age, gender, and comorbidities, which may influence PD-L1 expression differently within subgroups of smokers. While our data did not demonstrate a direct statistical significance, it opens a pathway for questioning the subtle and intricate ways in which smoking might influence tumor behavior and the efficacy of ICIs. For example, smoking has been shown to induce inflammatory responses and oxidative stress, which can lead to the activation of pathways that modulate PD-L1 expression on tumor cells (22). The lack of a direct correlation challenges the oversimplified view of smoking merely upregulating PD-L1 expression. Instead, it suggests a more nuanced interaction, possibly involving other immune regulatory pathways or alterations in the tumor microenvironment. Studies in other cancers, such as lung and head and neck cancers, have shown that smokers often exhibit higher levels of PD-L1. This increase is potentially due to chronic inflammatory responses induced by smoking, which may activate pathways like NF-kB and STAT3, key regulators of inflammation and immune response (23). This finding underscores the importance of considering multiple factors that can affect the tumor milieu and its interaction with the immune system (24), highlighting the need for a multidimensional approach in future research to unravel these complex relationships.

### Implications for personalized immunotherapy

The findings from our study advocate for a more individualized strategy in the use of ICIs, emphasizing the role of smoking history as a potential predictive factor in the absence of extensive genomic profiling. Recognizing that comprehensive genomic analysis is not always feasible in every clinical context, particularly due to resource constraints, a detailed smoking history could offer a valuable, readily obtainable indicator of a patient’s potential response to immunotherapy, especially when considering NMB. This approach gains even more significance considering the logistical and financial challenges associated with thorough NMB assessment (25). Our study not only contributes to the growing body of evidence supporting personalized medicine but also highlights the practical challenges and possibilities in implementing such approaches in diverse clinical settings. This serves as a call to action for the integration of more accessible and cost-effective markers (26), like smoking history, in the decision-making process for immunotherapy, potentially enhancing the efficacy and cost-effectiveness of cancer treatments.

### Future research directions

Our study opens several avenues for future research. Prospective studies are essential to validate the predictive value of smoking history in immunotherapy response. Additionally, it is crucial to explore the biological mechanisms underpinning the relationship between smoking-induced mutational changes and enhanced immunogenicity observed in former smokers. Investigating the potential interactions between smoking history, PD-L1 expression, and other molecular markers could yield a more comprehensive understanding of how to optimize immunotherapy for bladder cancer patients.

### The importance of cessation in the context of smoking and cancer

While our study provides valuable insights into the complex relationship between smoking history and the effectiveness of immunotherapy in bladder cancer, it is imperative to emphasize that these findings should not be misconstrued as an endorsement of smoking in any context. The association between former smokers and a distinct response to immunotherapy highlights an intriguing area of research, yet this observation must be balanced against the well-documented and significant risks associated with smoking. Smoking is a major risk factor for the development of bladder cancer and various other malignancies, and its detrimental effects on overall health are irrefutable (27).

It is essential to reiterate that smoking cessation is a critical component of cancer prevention strategies (28). Quitting smoking not only reduces the risk of developing cancer but also offers numerous other health benefits, contributing to overall wellbeing and quality of life (29). This study underscores the importance of cessation and reinforces the message that stopping smoking is beneficial for cancer prevention and health improvement.

Furthermore, our findings invite further research into how changes in lifestyle factors, like smoking, can influence cancer treatment outcomes, particularly in the realm of immunotherapy. Understanding these relationships could provide valuable insights into tailoring treatment strategies to individual patients, emphasizing the role of comprehensive patient history in informing therapeutic decisions. However, these insights should be leveraged to enhance personalized medicine and not to diminish the critical message that smoking cessation is essential for reducing cancer risk and improving health outcomes.

Nevertheless, it is important to acknowledge several limitations of our study. First, our reliance on self-reported smoking history may introduce bias due to potential inaccuracies in recall. To overcome this limitation, future studies could incorporate objective measures such as biomarkers for nicotine to validate these self-reports. Second, the retrospective design of our study limits our ability to control for all potential confounding factors and establish causality. Prospective studies are therefore recommended to provide a more robust framework to confirm our findings and explore the causative links further. Lastly, the absence of external validation could affect the generalizability of our results. We suggest that future research efforts focus on validating our findings across diverse populations and settings to enhance their robustness and applicability. Additionally, the inclusion of other biomarkers beyond PD-L1 could help elucidate the complex interactions within the tumor microenvironment and further validate the influence of smoking history on immunotherapy outcomes.

## Conclusion

In conclusion, our findings highlight the potential impact of smoking history on immunotherapy outcomes in bladder cancer patients. To build upon these insights, future research could beneficially focus on two key areas: first, a more in-depth investigation into the effects of smoking cessation timing relative to immunotherapy commencement; and second, a broader examination of how other lifestyle factors, in conjunction with smoking habits, may influence treatment efficacy. These focused areas of research could significantly contribute to the development of more personalized and effective treatment strategies for bladder cancer patients.

## Data availability statement

The original contributions presented in the study are included in the article/**Supplementary Materials**, further inquiries can be directed to the corresponding author/s.

## Ethics statement

The studies involving humans were approved by the ethics committee/institutional review board of Sun Yat-sen Memorial Hospital. The studies were conducted in accordance with the local legislation and institutional requirements. Written informed consent for participation in this study was provided by the participants’ legal guardians/next of kin.

## Author contributions

JK: Conceptualization, Formal analysis, Funding acquisition, Investigation, Project administration, Validation, Writing – original draft. YZ: Data curation, Writing – original draft. HZ: Data curation, Formal analysis, Methodology, Writing – review & editing. YH: Data curation, Formal analysis, Visualization, Writing – original draft. YL: Investigation, Software, Validation, Writing – review & editing. SF: Investigation, Methodology, Writing – review & editing. ZC: Data curation, Methodology, Validation, Writing – review & editing. JZ: Investigation, Methodology, Validation, Writing – review & editing. YQH: Data curation, Methodology, Writing – review & editing. ZS: Supervision, Validation, Visualization, Writing – review & editing. WX: Conceptualization, Methodology, Project administration, Writing – review & editing. XF: Writing – original draft, Writing – review & editing.
