# Supplementary material for: Assessing the predictive value of smoking history for immunotherapy outcomes in bladder cancer patients
Source: Front Immunol. 2024 Jun 13;15:1404812. doi: 10.3389/fimmu.2024.1404812 (PMC11208302; doi:10.3389/fimmu.2024.1404812)
Supplement: Supplementary file 1 [file DataSheet_1.pdf]

SUPPLEMENTARY MATERIALS

Supplementary Tables

Supplementary Table 1. Best overall response by smoking status

Best overall survival	Never Smoker (n=116)		Former Smoker (n = 197)		Current Smoker (n = 35)	
	n	%	N	%	n	%
Complete response	9	7.8	15	7.6	1	2.9
Partial response	11	9.5	29	14.7	3	8.6
Stable disease	20	17.2	36	18.3	7	0.20
Progressive disease	58	0.50	88	44.7	21	0.60
Not evaluated	18	15.5	29	14.7	3	8.5
Objective response rate (%)	17.2		22.3		11.4	

Supplementary Table 2. Univariate analysis of related clinical variables

Variable		N	Hazard ratio	p
**Surv ~ ECOG**				
**ECOG**		348	1.46 (1.25, 1.69)	<0.001
**Surv ~ Intravesical BCG administered**				
**Intravesical BCG administered**	N	265	Reference	
	Y	83	0.98 (0.73, 1.32)	0.89
**Surv ~ Met**				
**Met**	Liver	98	Reference	
	LN Only	60	0.35 (0.23, 0.54)	<0.001
	unknown	32	0.28 (0.16, 0.51)	<0.001
	Visceral	158	0.61 (0.46, 0.81)	<0.001
**Surv ~ NMB**				
**NMB**		348	0.78 (0.69, 0.88)	<0.001
**Surv ~ Received platinum**				
**Received platinum**	N	76	Reference	
	Y	272	1.41 (1.01, 1.98)	0.05
**Surv ~ Sex**				
**Sex**	F	76	Reference	
	M	272	0.81 (0.60, 1.10)	0.18
**Surv ~ TCGA Subtype**				
**TCGA Subtype**	I	118	Reference	
	II	95	0.96 (0.69, 1.35)	0.83
	III	69	1.23 (0.86, 1.75)	0.25
	IV	66	0.98 (0.68, 1.42)	0.92
**Surv ~ Tobacco Use History**				
**Tobacco Use History**	CURRENT	35	Reference	
	NEVER	116	0.98 (0.62, 1.53)	0.92
	PREVIOUS	197	0.88 (0.57, 1.34)	0.54

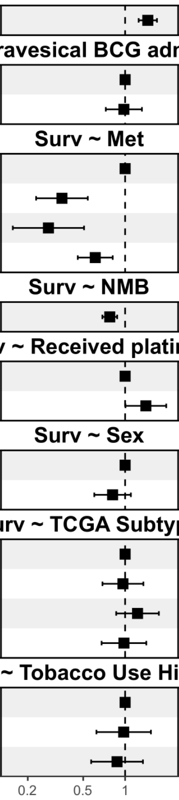

ECOG PS = Eastern Cooperative Oncology Group Performance Status;

Supplementary Table 3. Multivariate analysis of related clinical variables

Variable		N	Hazard ratio		p
**Surv ~ Sex + Intravesical BCG administered + ECOG + Received platinum + NMB + Tobacco Use Histo**					
**Sex**	F	76		Reference	
	M	272		0.84 (0.62, 1.15)	0.28
**Intravesical BCG administered**	N	265		Reference	
	Y	83		0.81 (0.59, 1.11)	0.20
**ECOG**		348		1.61 (1.35, 1.91)	<0.001
**Received platinum**	N	76		Reference	
	Y	272		1.59 (1.10, 2.30)	0.01
**NMB**		348		0.75 (0.66, 0.85)	<0.001
**Tobacco Use History**	CURRENT	35		Reference	
	NEVER	116		1.10 (0.69, 1.75)	0.68
	PREVIOUS	197		1.13 (0.73, 1.76)	0.59
**Met**	Liver	98		Reference	
	LN Only	60		0.45 (0.29, 0.70)	<0.001
	unknown	32		0.36 (0.20, 0.66)	<0.001
	Visceral	158		0.72 (0.53, 0.96)	0.03
**TCGA Subtype**	I	118		Reference	
	II	95		1.30 (0.91, 1.86)	0.14
	III	69		1.28 (0.88, 1.85)	0.20
	IV	66		0.87 (0.59, 1.27)	0.47

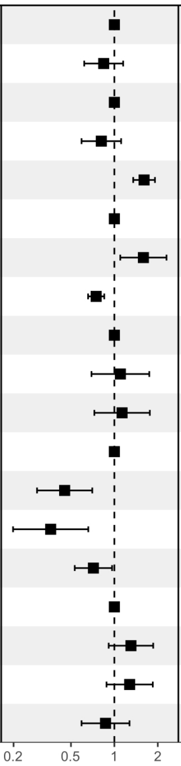

Supplementary Figures

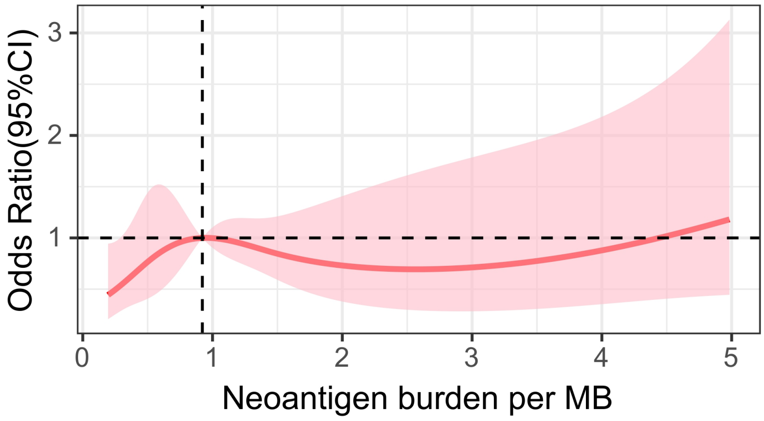

Supplementary Figure 1. Neoantigen Burden per MB and Risk of Immunotherapy Response. This graph illustrates the complex, non-linear association between the quantity of neoantigens per megabase (MB) in the tumor genome and the patient's risk of responding to immunotherapy. The curve depicts how variations in neoantigen burden influence the likelihood of a therapeutic response, highlighting key inflection points and thresholds.

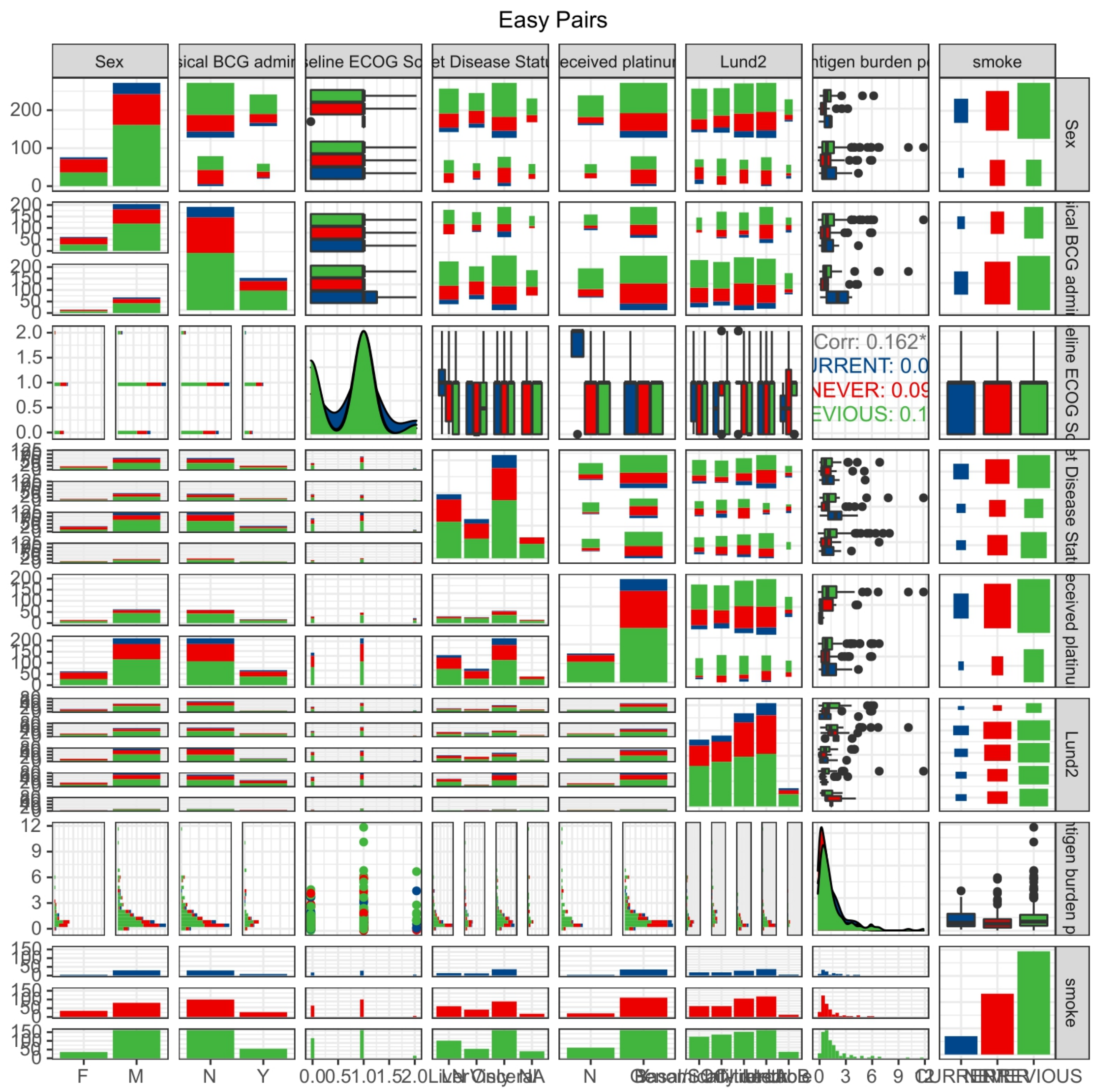

Supplementary Figure 2. Correlation matrix between independent variables

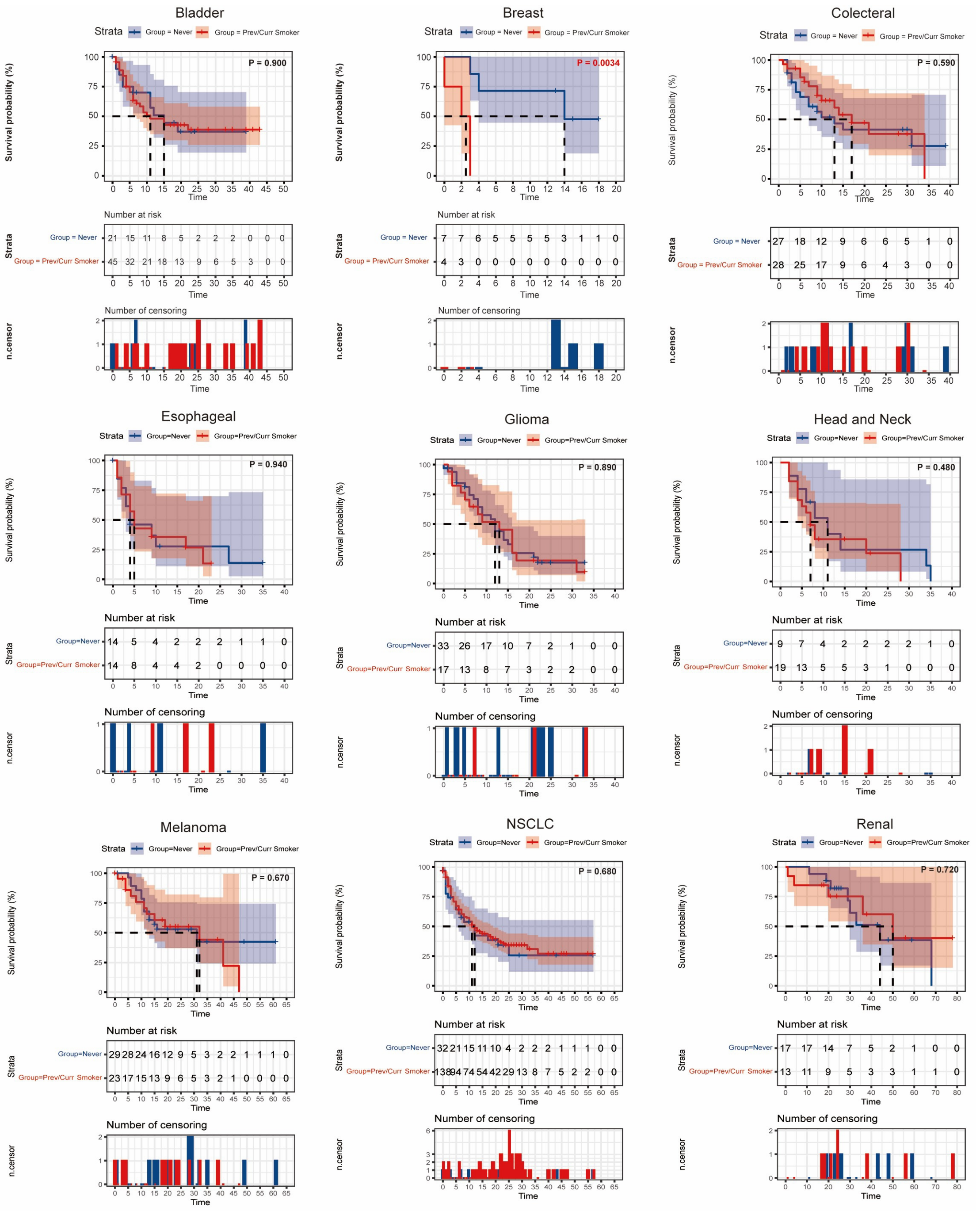

Supplementary Figure 3. Survival Outcomes by Smoking Status Across Various Cancers from the TCGA Database. This figure presents survival curves comparing the prognoses of patients with different cancers based on their smoking status, as sourced from the TCGA (The Cancer Genome Atlas) database. Notably, the survival impact of smoking is more pronounced in breast cancer patients, suggesting a significant correlation between smoking and survival in this group. In contrast, for other cancers represented, smoking status appears to have a less substantial effect on patient survival.
